# Impact of acromegaly subtypes on survival: results from a large multicenter cohort

**DOI:** 10.1210/jendso/bvag111

**Published:** 2026-05-13

**Authors:** Daniel Cuevas-Ramos, Artak Labadzhyan, Daniel Gomez, Rachel Fox, Froylan David Martínez-Sánchez, Andrea Rocha-Haro, Francisco J Gómez-Pérez, Shlomo Melmed

**Affiliations:** Neuroendocrinology Clinic, Department of Endocrinology and Metabolism, Instituto Nacional de Ciencias Médicas y Nutrición Salvador Zubiran, Mexico City, 14080, Mexico; Pituitary Center, Cedars-Sinai Medical Center, Los Angeles, CA 90048, USA; Pituitary Center, Cedars-Sinai Medical Center, Los Angeles, CA 90048, USA; Pituitary Center, Cedars-Sinai Medical Center, Los Angeles, CA 90048, USA; Neuroendocrinology Clinic, Department of Endocrinology and Metabolism, Instituto Nacional de Ciencias Médicas y Nutrición Salvador Zubiran, Mexico City, 14080, Mexico; Neuroendocrinology Clinic, Department of Endocrinology and Metabolism, Instituto Nacional de Ciencias Médicas y Nutrición Salvador Zubiran, Mexico City, 14080, Mexico; Neuroendocrinology Clinic, Department of Endocrinology and Metabolism, Instituto Nacional de Ciencias Médicas y Nutrición Salvador Zubiran, Mexico City, 14080, Mexico; Pituitary Center, Cedars-Sinai Medical Center, Los Angeles, CA 90048, USA

**Keywords:** growth hormone, pituitary adenoma, somatotroph, insulin-like growth factor 1

## Abstract

**Context:**

Acromegaly, characterized by excess growth hormone (GH) and insulin-like growth factor-1 production, is typically caused by a pituitary somatotroph adenoma. Disease activity and treatment responses vary widely according to its structural-functional classification comprising clinical, pathologic, morphologic, and biochemical features, particularly adenoma size and invasiveness on pituitary MRI and GH-granulation pattern.

**Objective:**

To evaluate the association between clinicopathologic acromegaly subtypes and long-term survival.

**Methods:**

This multicenter, bidirectional cohort study assessed all-cause mortality in adults with somatotroph adenomas classified into 3 different subtypes. Patients with Type 1 acromegaly have noninvasive or invasive microadenomas that are densely granulated; Type 2 noninvasive macroadenomas are densely or sparsely granulated; and Type 3 invasive macroadenomas are sparsely granulated. The primary outcome was all-cause mortality, analyzed by subtype.

**Results:**

The cohort comprised 550 patients, including 50.5% women, with a mean age at diagnosis of 42.3 years (standard deviation 13.7) and a median follow-up after diagnosis of 11.3 years (interquartile range 4.1-19.8). 172 patients (31.2%) had Type 1, 143 (26%) Type 2, and 235 (42.7%) Type 3. Overall mortality was 12.9%. Rates varied significantly across subtypes (*P* = .003): Type 1 had the lowest mortality at 7.0%, followed by Type 2 at 12.0%, and Type 3 at 17.9%. On multivariate Cox regression, Type 2 (hazard ratio [HR] = 2.76, 95% CI: 1.86-7.81, *P* = .009), and Type 3 (HR = 4.69, 95% CI: 1.65-13.3, *P* = .004) exhibited significantly higher mortality risk vs Type 1, independent of treatment modalities and presence of comorbidities.

**Conclusion:**

Applying a structural-functional acromegaly classification enables distinction of significant differences in long-term survival outcomes.

Pituitary adenomas exhibit distinctive clinical phenotypes determined by the adenohypophyseal cell of origin, and within these types, further adenoma differentiation leads to specific disease subtypes [1]. Adenomas giving rise to growth hormone (GH) hypersecretion derive from poorly or well differentiated somatotroph cells exhibiting varying degrees of GH granularity, which correlates with disease severity [2]. These adenomas are also associated with excess production of insulin-like growth factor 1 (IGF-1) [1, 3-5], leading to acromegaly features. Although the disorder is quite heterogenous, distinct clinical, pathologic, morphologic, and biochemical features have enabled disease subclassifications [6-9]. Diagnosis is often delayed for 10 or more years due to the heterogenous nature of insidious and nonspecific initial symptoms [10], leading to cardiovascular, cerebrovascular, metabolic, and respiratory comorbidities [11, 12] due to the prolonged uncontrolled GH secretion, with reduced life expectancy and a diminished quality of life [13-16].

We previously identified prognostic factors for acromegaly outcome prediction, including adenoma size, extrasellar invasiveness, adenoma granulation pattern, and immunohistochemical expression of p21 and somatostatin receptor Type 2 (SST2) [6, 17]. Based on these factors, we now portray a structural-functional classification to stratify somatotroph adenomas into 3 clinically distinct types. Type 1 acromegaly is associated with densely granulated microadenomas, which may or may not exhibit cavernous sinus invasion. These adenomas typically show strong SST2 and p21 expression and are associated with a favorable response to somatostatin receptor ligands (SRLs) and a favorable prognosis [6, 18, 19]. In Type 2 acromegaly, adenomas are densely or sparsely granulated macroadenomas without invasive features. These adenomas demonstrate intermediate biological behavior, treatment response, and clinical prognosis [6]. In Type 3 acromegaly, adenomas are invariably invasive, sparsely granulated macroadenomas. These often exhibit weak or absent SST2 and p21 expression [6], and have poorer response to standard treatments and more adverse clinical outcomes [20-23].

In clinical practice, some of these individual factors are often used to predict treatment outcomes. For example, adenoma size and invasion are used as determinants of surgical outcomes [9], and granulation pattern is used as a determinant of SRL responsiveness [4, 24]. However, whether specific clusters of these markers independently predict mortality remains unknown. Furthermore, earlier diagnosis of acromegaly and timely treatment can reduce complications and decrease mortality [25-27]. Yet, duration of disease control and the intensity of treatment required, including surgery, medications, and/or radiotherapy, can vary widely between acromegaly subtypes. As these differences may affect long-term prognosis and survival, we aimed to assess mortality in a large cohort of patients with acromegaly based on their disease subtype.

## Materials and methods

This multicenter, bidirectional cohort study evaluated all-cause mortality in patients classified in 1 of 3 distinct structural-functional subtypes. The study enrolled patients from the Neuroendocrinology Clinic, Department of Endocrinology, at the Instituto Nacional de Ciencias Médicas y Nutrición Salvador Zubirán (INCMNSZ) in Mexico City, Mexico, and from the Pituitary Center at Cedars-Sinai Medical Center in Los Angeles, CA, USA. These high-volume pituitary disease referral centers diagnose and manage patients with acromegaly in accordance with current international guidelines [28, 29]. The study protocol was approved by the Research Ethics Committee of both institutions: the INCMNSZ committee (#5188), and the Cedars-Sinai Institutional Review Board (#2873 and #2939). Written informed consent was obtained from all patients prior to their participation in the study.

### Participants

Utilizing a bidirectional cohort design, we first retrospectively abstracted clinical data from electronic medical records at both institutions, then prospectively traced and reassessed participants via direct contact during the follow-up phase. We included adult patients ≥18 years with a confirmed diagnosis of acromegaly, based on MRI evidence of adenoma and elevated serum IGF-1 levels, and/or a lack of GH suppression during a 75-gram oral glucose tolerance test (OGTT) [29]. Patients with non-GH-secreting pituitary adenomas or with adenomas exhibiting co-secretion of other hormones (eg, prolactin) [30], as well as those with familial genetic syndromes or multiple endocrine neoplasia [31] were excluded.

### Classification

Patients were categorized into 1 of 3 previously defined structural-functional subtypes based on MRI and pathology findings [6]. Patients were classified with Type 1 acromegaly if they had a microadenoma (<10 mm in its largest dimension) with a dense GH-granulation pattern on immunohistochemistry and presence or absence of cavernous sinus invasion and/or encasement of the internal carotid artery. Patients were classified with Type 2 acromegaly if they had a noninvasive macroadenoma, either densely or sparsely granulated, or with Type 3 acromegaly if they had an invasive macroadenoma with a sparsely granulated pattern. Adenomas were categorized as sparsely or densely granulated GH-expressing tumors using CAM5.2 cytokeratin immunostaining. A mixed granulation pattern, described as >30% tumor cells deviating from the dominant CAM5.2 pattern [32], is not separately described in pituitary adenoma classification [33] and acromegaly management consensus recommendations [28], and is therefore not included in the prognostic model. Invasiveness was assessed by pituitary MRI performed using 1.5T to 3T scanners with standardized sellar protocol and dynamic contrast-enhanced T1-weighted sequences to optimize adenoma detection.

### Data collection

Demographic, clinical, biochemical, imaging, and treatment-related data were extracted. The variables collected included age at diagnosis; prediagnosis disease duration (ie, age at diagnosis minus age at symptom onset); comorbidities (hypertension, diabetes mellitus, and cardiovascular disease); pituitary hormone levels at diagnosis (including GH, IGF-1 index [IGF-1 level divided by the upper limit of normal for age], and prolactin); adenoma characteristics on imaging and pathology; and history of treatment modalities (surgery, medical therapy, and radiotherapy). Mortality data were obtained through a comprehensive review of medical records, direct contact with patients or their relatives, and institutional mortality registries.

### Primary outcome

The primary outcome was all-cause mortality. Follow-up time was defined as the interval from diagnosis to the date of death or the last documented clinical follow-up. Cause-specific mortality was assessed as a secondary outcome.

### Statistical analysis

Data distribution was determined by Kolmogorov–Smirnov test. Normally distributed data were expressed as means ± standard deviation (SD), and skewed variables were expressed using median and interquartile ranges (IQR). Acromegaly subtypes were previously defined using a two-step model-based cluster analysis [5]. Discriminant analysis tested the accuracy of patient classification and overlap between groups. Bivariate comparisons were performed using the Chi square test, independent Student's *t*-test, or the Mann–Whitney *U*-test, as appropriate. Differences in quantitative variables across acromegaly types were assessed using one-way ANOVA. Bivariate analyses were performed across acromegaly subtypes using the Bonferroni correction to account for multiple comparisons. Linear trends between groups were confirmed using one-way ANOVA. The probability of mortality across types was evaluated with Kaplan–Meier survival analysis and compared using the log-rank test. Multivariate Cox proportional hazards models were used to estimate adjusted hazard ratios (HR) for mortality by acromegaly type, controlling for potential confounding variables, including age, sex, comorbidities, IGF-1 and GH levels at diagnosis, and treatment modalities. Statistical significance was defined as a two-sided *P* value <.05. All analyses were conducted using IBM SPSS Statistics version 26.0.

## Results

### Baseline characteristics

**Figure 1:**
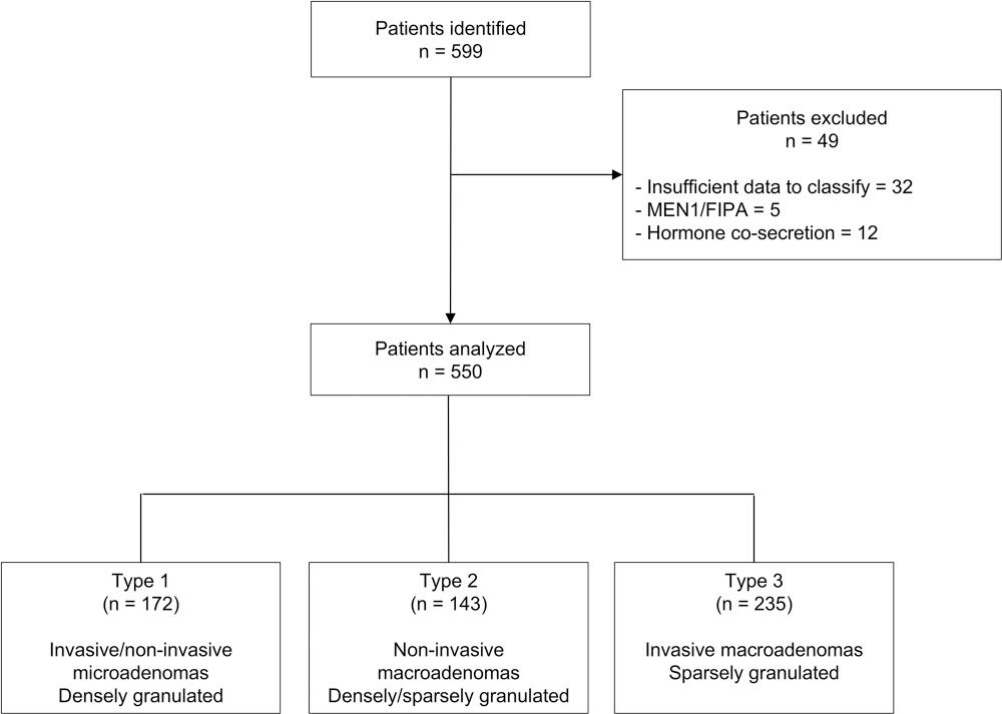
Flow chart of the study population. FIPA, familial isolated pituitary adenoma; MEN1, multiple neoplasia Type 1.

A total of 599 patients with acromegaly were identified; 49 (8.2%) were excluded because of missing data, familial syndromes, or cosecreting adenomas. Therefore, the final cohort comprised 550 patients. Of these, 292 patients were previously reported [6] and classified as follows: Type 1, n = 147; Type 2, n = 55; and Type 3, n = 90. An additional 258 patients were newly identified and classified as follows: Type 1, n = 25; Type 2, n = 88; Type 3, n = 145. Therefore, in the final cohort, distribution was as follows: Type 1, n = 172 (31.3%); Type 2, n = 143 (26.0%); and Type 3, n = 235 (42.7%) (Fig. 1).

The mean age at diagnosis was 42.5 (SD 13.7) years, and 50.5% of patients were female. The median disease duration before diagnosis was 5.7 years (IQR 2.3-10.2), and median follow-up time after diagnosis was 11.3 years (IQR 4.2-19.8) (Table 1). A total of 68.7% (n = 378) patients had macroadenomas, with a median size of 14 mm (IQR 10-21); on histology, 42% (n = 232) of adenomas were densely granulated and 57.8% (n = 318) were sparsely granulated. Cavernous sinus invasion was documented in 48.9% (n = 269). Median IGF-1 level at diagnosis was 675 ng/mL (IQR 431-921), with a corresponding median IGF-1 index of 2.5 (IQR 1.6-3.7). Overall, 24% of patients had diabetes mellitus, 34% hypertension, and 18% colon polyps.

**Table 1:** Clinical, biochemical, and adenoma-related characteristics by acromegaly subtype

		Acromegaly classification			
	All (n = 550)	Type 1 (n = 172)	Type 2 (n = 143)	Type 3 (n = 235)	*P* value
Demographics					
Age, years, mean (SD)	42.3 (13.7)	48.6 (13.1)	41.5 (12.3)	39.0 (13.4)	<.001
Female, n (%)	278 (50.5)	85 (49.4)	74 (51.7)	119 (50.6)	.918
Prediagnosis disease duration, years, median (IQR)	5.7 (2.3-10.2)	7.2 (3-12)	6 (2.4-10.4)	4.7 (1.8-9.2)	.026
Follow-up, years median (IQR)	11.3 (4.1-19.8)	13.2 (7.2-20.7)	11.6 (6.0-19.0)	9.3 (3.0-19.4)	.002
Overall mortality	71 (12.9)	12 (7.0)	17 (12.0)	42 (17.9)	.003
Comorbidities					
Hypertension, n (%)	186 (34)	74 (43)	39 (27)	73 (31)	.008
Diabetes mellitus, n (%)	135 (24)	48 (28)	28 (20)	59 (25.1)	.219
Colon polyps, n (%)	99 (18)	26 (15)	25 (17.5)	48 (20)	.203
Adenoma characteristics					
Microadenomas, n (%)	172 (31.3)	172 (100)	0 (0.0)	0 (0.0)	<.001
Macroadenomas, n (%)	378 (68.7)	0 (0.0)	143 (100)	235 (100)	<.001
Size mm, median (IQR)	14 (10-21)	7.6 (6.0-9.9)	16.3 (11-22)	18 (14-25)	<.001
Cavernous sinus invasion, n (%)	269 (48.9)	34 (32)	0 (0.0)	235 (100)	<.001
Densely granulated, n (%)	232 (42)	172 (100)	60 (42.0)	0 (0.0)	<.001
Sparsely granulated, n (%)	318 (57.8)	0 (0.0)	83 (58)	235 (100)	<.001
Biochemistry at diagnosis					
IGF-1 ng/mL, median (IQR)	675 (431-921)	608 (384-836)	641 (418-866)	746 (472-999)	.002
IGF-1 index*^a^*, median (IQR)	2.5 (1.6-3.7)	2.4 (1.6-3.4)	2.5 (1.6-3.4)	2.8 (1.7-4.0)	.106
GH ng/mL*^b^*, median (IQR)	15 (7-32)	11 (6-22)	14 (7-37.2)	18 (9.5-41)	.001
PRL ng/mL, median (IQR)	15.7 (8-53)	12 (7.6-23)	16 (8.5-59)	18.5 (9-62)	.015

Abbreviations: GH, growth hormone; IGF-1, insulin-like growth factor 1; PRL, prolactin.

^*a*^IGF-1 divided by the upper limit of normal for age.

^*b*^Measured during a 75-gram OGTT.

A total of 86.5% of patients underwent surgery; 68.5% had a single surgical procedure, 14.7% had 2, and 2.2% had 3 or more surgical interventions (Table 2). Of the 52.5% of patients receiving medical treatment, 34% were treated with dopamine receptor agonists, 34% with SRLs, and 6.1% with pegvisomant. Of the 25% of patients who received radiotherapy, 19.3% were treated with conventional and 5.8% with stereotactic radiotherapy.

**Table 2:** Treatment modalities by acromegaly subtype

	Acromegaly classification				
	All (n = 550)	Type 1 (n = 172)	Type 2 (n = 143)	Type 3 (n = 235)	*P* value
Medical therapy, n (%)					
Somatostatin receptor ligands	187 (34)	46 (26.7)	37 (26)	104 (44)	.003*^a^*
Dopamine agonists	186 (34)	44 (25.5)	45 (31.5)	97 (41.2)	.01*^b^*
Pegvisomant	34 (6.1)	4 (2.3)	8 (5.6)	22 (9.3)	.02*^b^*
Number of medical therapies*^c^*, n (%)					<.001*^b^*
None	261 (47.4)	99 (57.5)	76 (53.1)	86 (36.5)	
1	177 (32.2)	47 (27.3)	45 (31.4)	85 (36.1)	
2	88 (16)	22 (12.8)	18 (12.6)	48 (20.4)	
3	18 (3.2)	1 (0.6)	3 (2.1)	14 (6.0)	
Number of surgeries, n (%)					.02*^a^*
None	74 (13.5)	23 (13.4)	21 (14.6)	30 (1.3)	
1	377 (68.5)	127 (73.8)	96 (67.1)	154 (65.5)	
2	81 (14.7)	16 (9.3)	24 (16.7)	41 (17.4)	
3 or more	12 (2.2)	3 (1.7)	1 (0.7)	8 (3.4)	
Radiotherapy, n (%)					.01*^a^*
None	337 (61.3)	104 (60.4)	89 (62.2)	144 (61.2)	
Conventional	106 (19.3)	25 (14.5)	32 (22.3)	49 (20.8)	
Stereotactic	32 (5.8)	4 (2.3)	7 (4.9)	21 (8.9)	
Number of treatment modalities, n (%)					<.001*^a^*
None	33 (6)	10 (5.8)	6 (4.2)	17 (7.2)	
1	184 (33.4)	83 (48.2)	52 (36.3)	49 (20.8)	
2	145 (26.3)	37 (21.5)	45 (31.4)	63 (26.8)	
3	99 (18)	26 (15.1)	25 (17.4)	48 (20.4)	
4	51 (9.2)	6 (3.5)	11 (7.7)	34 (14.4)	
5 or more	34 (6.1)	8 (4.6)	4 (2.8)	22 (9.3)	
Combination treatment modalities, n (%)					<.001*^a^*
Surgery + medical therapy	172 (31.2)	45 (26.1)	36 (25.1)	91 (38.7)	
Surgery + radiotherapy	51 (9.3)	13 (7.5)	16 (11.2)	22 (9.3)	
Surgery + medical therapy + radiotherapy	73 (13.2)	13 (7.5)	16 (11.2)	44 (18.7)	

^*a*^χ^2^ test.

^*b*^Lineal by lineal χ^2^ test.

^*c*^SRLs and/or dopamine agonists and/or pegvisomant.

### Clinical and biochemical characteristics by acromegaly subtype

Analyzing clinical and biochemical characteristics in our overall cohort, we found significant differences between acromegaly subtypes (Table 1). Compared with patients with Types 2 and 3 acromegaly, those with Type 1 acromegaly were diagnosed at an older age (*P* < .001), had a longer disease duration before diagnosis (*P* = .026), and longer survival (*P* = .003). Type 1 patients also exhibited a higher frequency of hypertension (*P* = .008). By definition, all Type 1 patients had microadenomas, which corresponded with a smaller median adenoma size (*P* < .001), less prevalent invasiveness (*P* < .001), and a densely granulated histologic pattern (*P* < .001). At diagnosis, Type 1 acromegaly was associated with the lowest IGF-1 and GH levels, while Type 3 patients had the highest levels (*P* = .002 and *P* = .001, respectively).

### Treatment modalities by acromegaly subtype

We compared treatment modalities during follow-up by acromegaly type (Table 2). Patients with Type 3 acromegaly more frequently required more than one SRL, pegvisomant, and/or dopamine agonist than did patients with other types (*P* < .001). Dopamine agonists, SRLs, and radiotherapy were used more frequently in patients with Types 2 and 3; a higher proportion of these patients also required a combination of 2 or more treatment modalities to achieve disease control (*P* < .001) as well as more surgical procedures (*P* = .02). Across all groups, Type 1 patients had the lowest proportion receiving either conventional or stereotactic radiotherapy (*P* = .01). Furthermore, nearly half of Type 1 patients achieved disease control with only a single therapeutic modality, whereas patients in the Types 2 and 3 groups more frequently required 2 to 5 treatment modalities (*P* < .001).

### Mortality risk by acromegaly subtype

**Figure 2:**
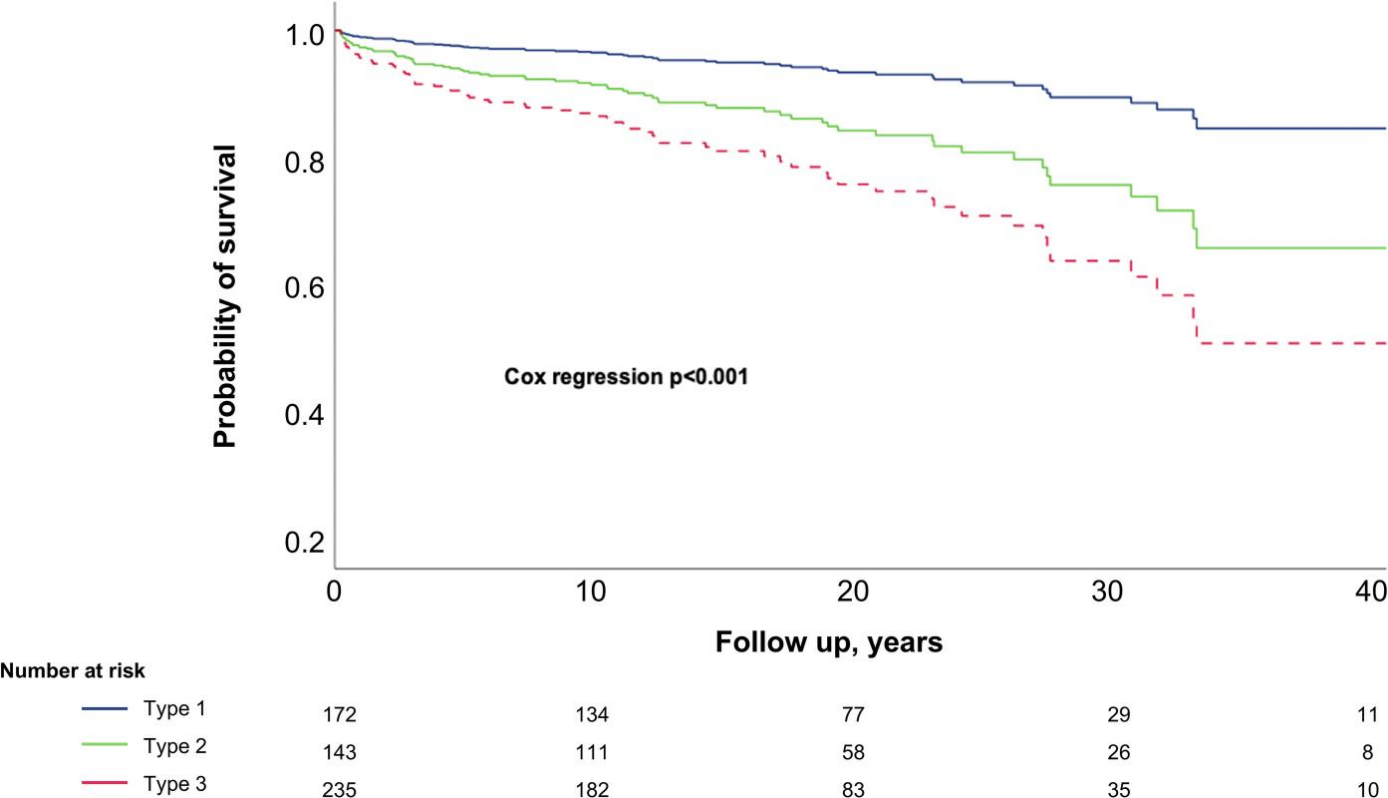
Probability of survival by acromegaly subtype. Type 1 acromegaly is shown in blue, Type 2 in green, and Type 3 in red.

Mortality rate in the entire cohort was 12.9% (n = 71); by subtype, rates were 7.0% (n = 12) for Type 1, 12.0% (n = 17) for Type 2, and 17.9% (n = 42) for Type 3 (*P* = .003). Probability of survival was significantly lower for Types 2 and 3 compared with Type 1 (*P* < .001). Notably, this survival disadvantage for Types 2 and 3 was apparent from the beginning of follow-up and was sustained, with the difference widening over time (Fig. 2). The consistent mortality trend across acromegaly subtypes was confirmed in the hospital-stratified analysis, with a statistically significant difference observed at each participating center (Cedars-Sinai Medical Center, *P* = .036; INCMNSZ, *P* = .037).

Unadjusted analysis demonstrated that both Types 2 and 3 were associated with a significantly higher mortality risk compared with Type 1 (HR = 3.09, 95% CI: 1.62-5.91, *P* < .001). We subsequently constructed 3 multivariate Cox regression models (Table 3). Model 1, which adjusted for age and sex, confirmed that Types 2 and 3 were significantly associated with increased mortality risk (*P* < .001). Model 2, which additionally adjusted for IGF-1 and GH levels, hypertension, and diabetes mellitus, indicated that Types 2 and 3 was associated with a 2- and 4-fold higher risk of death, respectively. Finally, Model 3, which additionally adjusted for the number of treatment modalities, showed that Type 3 patients had a 3-fold higher mortality risk, independent of the number of treatment modalities administered. Both older age and the presence of diabetes mellitus were also significantly and independently associated with a higher mortality risk in this group.

**Table 3:** Multivariate cox proportional hazards models for mortality by acromegaly subtype

	Hazard ratio	95% CI	*P* value
Model 1			
Type 1		1 (reference)	
Type 2	2.49	1.15-5.40	.02
Type 3	4.03	2.05-7.96	<.001
Age	1.05	1.02-1.07	<.001
Model 2			
Type 1		1 (reference)	
Type 2	3.19	1.15-8.82	.026
Type 3	5.29	2.11-13.2	<.001
Age	1.04	1.02-1.07	<.001
Diabetes mellitus	1.97	1.05-3.68	.03
Model 3			
Type 1		1 (reference)	
Type 2	2.76	1.86-7.81	.009
Type 3	4.69	1.65-13.3	.004
Age	1.04	1.02-1.07	<.001
Diabetes mellitus	2.13	1.06-4.26	.03

Abbreviation: CI, confidence interval. Only significant variables are shown.

Parameters of model 1: −2LL = 727.8; χ^2^ = 32.4, *P* < .001. Model 1 adjusted for age and sex.

Parameters of model 2: −2LL = 469.3; χ^2^ = 32.5; *P* < .001. Model 2 adjusted for model 1 plus hypertension, diabetes mellitus, and IGF-1, and GH levels at diagnosis.

Parameters of model 3: −2LL = 377.3; χ^2^ = 39.4; *P* = .004. Model 3 adjusted for model 2 plus dopamine agonists, somatostatin receptor ligands, pegvisomant, number of surgeries, and radiotherapy.

### Causes of mortality stratified by acromegaly type

The distribution of overall and cause-specific mortality varied significantly across acromegaly types (Table 4). The leading cause of death for all types was cardiovascular disease (4%, n = 22), which was significantly more frequent in Type 3 (*P* = .01). Cerebrovascular events (1.6%, n = 9) and pneumonia (1.5%, n = 8) also showed a clear preponderance in the Type 3 subtype. Mortality from cancer was low (1%, n = 6) and was distributed heterogeneously across groups. A total of 3.5% (n = 19) of deaths remained unclassified because of missing reliable data.

**Table 4:** Causes of death by acromegaly subtype

		Acromegaly classification			
	All (n = 550)	Type 1 (n = 172)	Type 2 (n = 143)	Type 3 (n = 235)	*P* value
Overall	71 (13.2)	12 (7)	17 (11.9)	42 (18.8)	.003*^a^*
Cardiovascular	22 (4.0)	3 (1.7)	6 (4.2)	13 (5.5)	.01*^b^*
Cerebrovascular	9 (1.6)		3 (2.0)	6 (2.5)	—
Pneumonia	8 (1.5)		1 (0.6)	7 (2.9)	—
Cancer	6 (1.2)	3 (1.7)	2 (1.3)	1 (0.4)	—
Sepsis	2 (0.4)	1 (0.58)		1 (0.4)	—
Chronic kidney disease	1 (0.2)			1 (0.4)	—
Acute lymphoblastic leukemia	1 (0.2)			1 (0.4)	—
Intestinal obstruction	1 (0.2)	1 (0.58)			—
Overdose	1 (0.2)			1 (0.4)	—
Parkinson disease	1 (0.2)	1 (0.58)			—
Unknown	19 (3.5)	3 (1.7)	5 (3.4)	11 (4.6)	—

Data expressed as n (%).

^*a*^Cox regression analysis.

^*b*^Lineal by lineal χ^2^ test.

## Discussion

This large bidirectional cohort study provides compelling evidence that the structural-functional classification of acromegaly delineates distinct clinical entities with type-specific differences in disease behavior, therapeutic requirements, and long-term survival. Our findings confirm that this stratification system is not solely a clinicopathologic descriptor, but also a helpful tool for mortality risk assessment that can be used as part of a personalized management approach by physicians caring for patients with acromegaly.

The demographic and clinical profile of our cohort is consistent with previous large-scale acromegaly studies, particularly the high prevalence of macroadenomas (68.7%) and cavernous sinus invasion (48.9%), underscoring the aggressive nature of many somatotroph adenomas [34]. The significant burden of cardiometabolic comorbidities, such as hypertension (34%) and diabetes mellitus (24%), aligns with the known systemic effects of chronic GH and IGF-1 excess [10, 12, 16, 35].

We observed that patients with Types 2 and 3 acromegaly were diagnosed at a significantly younger age and after a significantly shorter prediagnosis disease duration compared with patients who had Type 1 acromegaly. It is likely that the more adverse clinical severity of Types 2 and 3, including expanding effects from the macroadenoma mass, would have prompted earlier medical consultation leading to diagnosis at a younger age. These patients also had a shorter follow-up time, consistent with their subsequent higher mortality rate. We included age as a covariate in the multivariate Cox proportional hazards regression analysis to adjust for this potential confounding effect and still confirmed that Types 2 and 3 were significantly associated with increased mortality risk.

Indeed, we found a stark mortality gradient across the 3 acromegaly types. Patients with Type 3 acromegaly harboring sparsely granulated, invasive macroadenomas exhibited a mortality rate nearly 3-fold higher than that of Type 1 patients. This elevated risk persisted even after adjusting not only for age, but also for sex, cardiometabolic comorbidities, IGF-1 levels at diagnosis, and treatment modalities. Of note, the risk was independent of the number of treatments required, suggesting that the intrinsic biological aggressiveness of Type 3 tumors, rather than simply treatment resistance, is a key factor contributing to poor survival. Furthermore, the difference in mortality was evident even after stratifying by center, suggesting that any local differences in implementing management guidelines do not adversely affect survival. These observations reinforce the concept that acromegaly subtype is an independent prognostic factor, as suggested in our earlier, smaller series [6], and that it complements biomarker-driven, personalized treatment approaches for optimizing patient outcomes.

The distinctive cause-specific mortality offers insights into possible mechanisms underlying the disparity in overall mortality. The significantly higher rate of cardiovascular deaths in patients with Type 3 acromegaly is notable. While cardiovascular disease is a leading cause of death in acromegaly [25, 36-38], its overwhelming predominance in the Type 3 subgroup suggests that the more severe hormonal excess and treatment challenges in these patients lead to accelerate and less controllable cardiovascular damage. Similarly, the higher mortality from cerebrovascular events and pneumonia in Type 3 patients could reflect a more debilitated general state.

Type 1 acromegaly with densely granulated microadenomas consistently demonstrated the most indolent course. These patients presented with lower GH/IGF-1 levels, achieved disease control with fewer therapeutic modalities, and had significantly longer survival. This observation aligns with the understanding that patients with smaller adenomas have a higher probability of surgical success and that those with densely granulated adenomas often exhibit a more favorable response to SRLs [4, 19, 24]. Distribution of cancer-related mortality was more frequent in Types 1 and 2 patients compared with Type 3. While the overall cancer mortality rate was low (1.1%), it is possible that the longer survival time in patients with less aggressive Types 1 and 2 acromegaly provides a wider temporal window for development and manifestation of malignancies, a long-term concern in acromegalic patients due to the mitogenic potential of chronic GH and IGF-1 excess [36, 39-41].

Conversely, the shorter survival of Type 3 patients, attributable primarily to cardiovascular and cerebrovascular causes, may have resulted in a competing risk, wherein these patients succumbed more frequently to rapid metabolic complications before cancer could clinically manifest. Indeed, only 1 cancer-related death was reported among 42 fatalities in this subgroup. This consideration is important for interpreting cause-specific mortality data in acromegaly cohorts with different survival rates.

Our classification robustly confirms distinct disease behaviors across acromegaly subtypes that directly impact mortality risk. Notably, Type 1 adenomas were all densely granulated microadenomas, associated with milder symptomatology and disease activity, without conferring compressive effects. Interestingly, they persist as microadenomas despite longer disease duration prior to diagnosis, likely due to increased p21 expression and subsequent cell-cycle arrest and senescence features [6, 17]. By contrast, despite a shorter disease duration before diagnosis, Types 2 and 3 acromegaly present as macroadenomas with a higher symptom burden, more adverse disease activity, and central compressive effects. These findings align with the phenotypic heterogeneity that we previously described [6], and that we now prospectively validated as significant contributors to mortality risk.

Our results have direct clinical implications. Given the increased mortality risk and the aggressive nature of Types 2 and 3 acromegaly, a more proactive, multifaceted treatment approach is required from the outset. The fact that age and diabetes mellitus remained independent predictors of mortality across all models highlights the critical need for rigorous comorbidity management alongside adenoma-targeted therapy [28].

A limitation of this study is its partial reliance on retrospective data, which is common in research on rare diseases. However, combining these records with data from a prospective pituitary adenoma research registry enabled comprehensive follow-up and facilitated compilation of a large sample size. The number of patients excluded due to missing data was small, and is unlikely to have significantly altered the observed associations. The resultant robust dataset provided the statistical power to strengthen our conclusions through multivariate analysis. Adenoma invasion was defined using MRI. To mitigate potential interpretation bias, all MRI assessments underwent independent review by expert neuroradiologists blinded to the subtype classification at each participating center. Acromegaly subtype definitions comprise key pathologic, clinical, and imaging markers, but we cannot exclude that other factors, including cumulative IGF-1 exposure, may also contribute to mortality risk [40].

Acromegaly and pituitary adenoma classifications based on pathology or imaging have been proposed [8, 9] and there remains a need to consolidate clinical, imaging, pathologic and therapeutic outcomes into a comprehensive classification system that can be broadly applied in the clinic [7, 42]. Our study adds to this body of knowledge by offering a structural-functional classification that can predict mortality risk. In validating our classification schema, we provide a robust framework for stratifying patient care, optimizing resource allocation, and counseling individuals on their long-term prognosis and survival. Future research should focus on elucidating and consolidating molecular keystones of these subtypes to develop more effective, targeted therapies for high-risk patients.

## Data Availability

Restrictions apply to the availability of some or all data generated or analyzed during this study to preserve patient confidentiality. The corresponding author will on request detail the restrictions and any conditions under which access to some data may be provided.
